# Processing Suitability and Flavor Profiles of Wagyu Beef Tallow from Different Anatomical Regions

**DOI:** 10.3390/molecules31030426

**Published:** 2026-01-26

**Authors:** Yanxia Xing, He Zhu, Mengqi Li, Yanfei Yang, Mengliu Zhu, Yushu Wang, Zien Li, Baochen Xu, Yang Yu, Lizeng Peng

**Affiliations:** 1 College of Modern Beef Cattle Industry, Shandong Agriculture and Engineering University, Jinan 250100, China; z2014007@sdaeu.edu.cn (Y.X.); yanfei.yang.research@outlook.com (Y.Y.); z2021014@sdaeu.edu.cn (M.Z.); yushu.wang.science@hotmail.com (Y.W.); lizien.academic@foxmail.com (Z.L.); xvbaoo0@gmail.com (B.X.); 2 College of Food Science and Engineering, Qingdao Agricultural University, Qingdao 266109, China; 20252217022@stu.qau.edu.cn (M.L.); z2017025@sdaeu.edu.cn (Y.Y.); 3 Key Laboratory of Novel Food Resources Processing, Institute of Agro-Food Sciences and Technology, Shandong Academy of Agricultural Sciences, Jinan 250100, China

**Keywords:** Wagyu beef tallow, HS-SPME-GC-MS, PCA

## Abstract

This study investigated the technological properties and volatile flavor profiles of tallow from three anatomical regions of Wagyu cattle, omental fats (OF), perirenal fats (PF), and subcutaneous fats (SF), smelted at temperatures ranging from 100 to 160 °C. The objective was to provide a theoretical basis for the targeted utilization of Wagyu fats. Results showed that smelting temperature significantly affected oil yield, with the highest yield obtained at 160 °C for all regions. PF exhibited the greatest oil yield, followed by OF and SF. Physicochemical analyses indicated that OF had the highest degree of unsaturation, whereas PF demonstrated superior hardness and oxidative stability. Microstructural and spectroscopic analyses, Fourier-transform infrared (FTIR) spectroscopy and scanning electron microscopy (SEM) were employed to characterize the samples, revealed that the compact protein structure of SF residues limited oil release, while the porous structures of OF and PF residues facilitated higher yields. With respect to flavor profiling, headspace solid-phase microextraction coupled with gas chromatography–mass spectrometry (HS-SPME-GC-MS) was employed to analyze volatile compounds, identified aldehydes as the dominant flavor contributors in OF and PF, imparting fatty and citrus notes, whereas SF was characterized by a distinct creamy aroma primarily due to γ-butyrolactone. These regional differences were further validated by principal component analysis (PCA). Overall, PF obtained the highest comprehensive quality score. The integrated evaluation underscores the potential for precision-based utilization of Wagyu tallow: PF and OF are recommended for applications demanding high yield and intense flavor, whereas SF, characterized by its distinctive creamy aroma, is more suitable for specialized or niche products.

## 1. Introduction

Wagyu beef tallow possesses considerable application potential in the high-end food industry owing to its excellent oxidative stability and distinctive, rich flavor profile [1,2]. The complexity of its flavor arises from the synergistic effects of breed-specific genetics and specialized feeding practices, making Wagyu tallow a premium medium for enhancing both the sensory attributes and economic value of food products. However, current research lacks a systematic evaluation framework for assessing tallow derived from adipose tissues originating from different anatomical regions of Wagyu cattle.

This knowledge gap has resulted in the underutilization of high-value, region-specific tallow, which is frequently overlooked or discarded [3,4]. Such underuse not only leads to resource waste but also limits its further development for industrial applications, including hotpot bases and the flavor standardization of prepared foods. Although previous studies have compared the physicochemical properties [5] or volatile flavor components of fats obtained from different adipose depots [6], most investigations remain largely descriptive. Comprehensive research that integrates processing suitability parameters, such as oil yield, hardness, and oxidative stability, with systematic profiling of volatile flavor compounds remains scarce. More critically, the microstructural characteristics and protein conformational mechanisms underlying differences in processing performance among tallow from various anatomical regions, as well as the formation pathways of key aroma-active compounds associated with specific depots, have yet to be fully elucidated.

To address these gaps, the present study was designed with three primary objectives: (1) to systematically evaluate the processing suitability of Wagyu fats derived from three anatomical regions (omental, perirenal, and subcutaneous depots) under different rendering temperatures; (2) to elucidate microstructural alterations and protein secondary structure variations influencing oil yield using scanning electron microscopy (SEM) and Fourier-transform infrared (FTIR) spectroscopy; and (3) to characterize region-specific volatile flavor profiles and identify key aroma-active compounds using headspace solid-phase microextraction coupled with gas chromatography–mass spectrometry (HS-SPME-GC-MS). The findings of this study are expected to provide a theoretical basis for the targeted, value-added utilization of Wagyu tallow according to its anatomical origin.

## 2. Results and Discussion

### 2.1. Oil Yield

As presented in Table 1, the liquid fraction of beef tallow increased with rising smelting temperature, reaching its maximum yield at 160 °C. Among the samples, PT exhibited the highest yield (72.42%), followed by OT (64.58%) and ST (59.03%). Significant differences (*p* < 0.05) were observed among the three fractions, with particularly pronounced variations between ST and both OT and PT. The residue-to-fat ratio (g residue/g fat) serves as a direct indicator of rendering efficiency, whereby higher values reflect less complete fat release from the adipose tissue matrix. The lower rendering efficiency observed for ST is likely attributable to its higher connective tissue content, which forms a denser collagenous network that acts as a physical barrier, thereby hindering the flow and coalescence of molten fat during heating [7]. These marked regional differences in oil yield can be explained by distinct tissue compositions and microstructural characteristics, as demonstrated in the subsequent analyses. Specifically, the reduced oil yield of ST is associated with its greater connective tissue content and more compact microstructure, which physically restrict the release and migration of lipid droplets during the rendering process. In contrast, the looser and more porous microstructures observed in OT and PT residues facilitate more efficient oil flow and fat recovery. This interpretation is consistent with the findings of Yamada et al. [8] regarding depot-specific fat characteristics and is further supported by the greater stability of protein secondary structures detected in ST residues. Such structural stability may contribute to the persistence of a restrictive tissue matrix during thermal processing, thereby limiting fat liberation.

### 2.2. IVs

**Figure 1:**
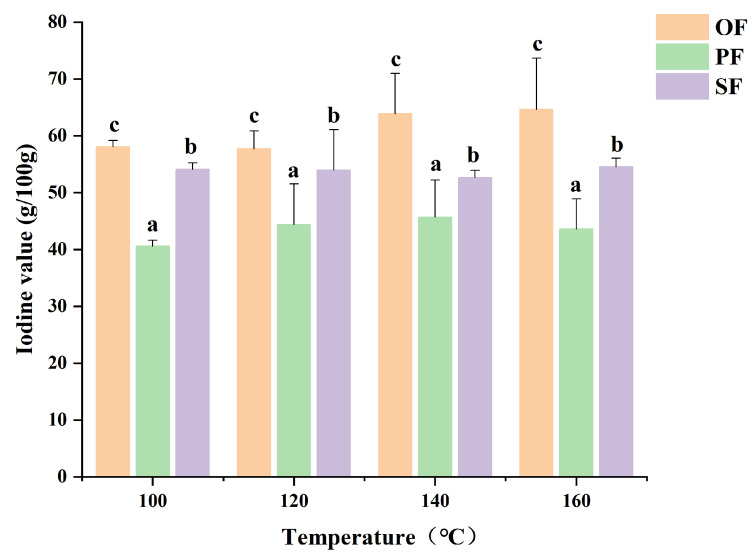
*IVs* of Wagyu beef tallow from different parts at various smelting temperatures. a~c indicates the difference between different sites is significant (*p* < 0.05). OF, omental fats; PF, perirenal fats; SF, subcutaneous fats.

Fatty acids with higher saturation levels generally display lower iodine values (*IVs*), reflecting a reduced degree of unsaturation [9]. As illustrated in Figure 1, smelting temperature showed no significant effect on the *IVs* of the liquid oil across different parts. However, notable variations were observed among the different anatomical regions. Among the samples, the liquid oil from OF exhibited the highest *IV*, indicating a greater abundance of unsaturated fatty acids and consequently higher oxidative sensitivity [10]. In contrast, the liquid oil from PF consistently presented significantly lower *IV* than those from other parts, implying a lower concentration of unsaturated fatty acids. This property suggests that the PF liquid oil demonstrates stronger oxidative stability, making it more suitable for prolonged storage and transportation. Additionally, variations in *IVs* were found to correlate with differences in the flavor profile of the liquid oil [11].

### 2.3. Hardness

**Figure 2:**
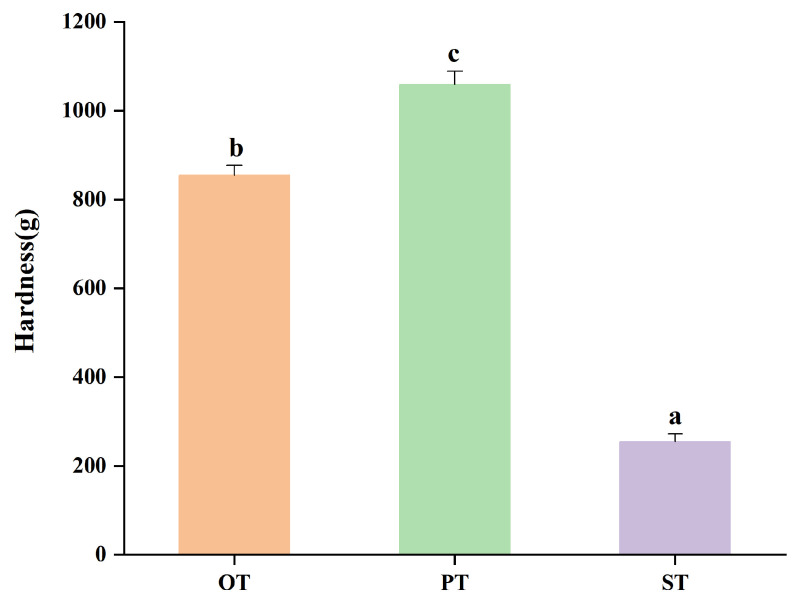
The hardness of different parts of tallow at 4 °C. a~c indicates the difference between different sites is significant (*p* < 0.05). OT, omental tallow; PT, perirenal tallow; ST, subcutaneous tallow.

The hardness of the tallow samples is presented in Figure 2; the data of duplicate samples are listed in Table S1. A clear increasing trend in hardness was observed from ST to OT and ultimately to PT. Among the three fractions, PT exhibited the highest hardness value (1058.97 g), which was significantly greater than those of OT and ST (*p* < 0.05). The relatively low standard deviation (SD = 20.03) obtained from the hardness measurements indicates a uniform texture and minimal internal variability among the samples. This regional gradation in hardness is consistent with the characteristic texture range reported for industrial tallow products [7] and is likely associated with differences in compositional properties. In particular, PT exhibited the lowest IV, indicating a lower degree of unsaturation, which is commonly associated with increased fat firmness and structural rigidity.

### 2.4. FTIR Analysis of Chemical Composition and Protein Structure in Residues

**Figure 3:**
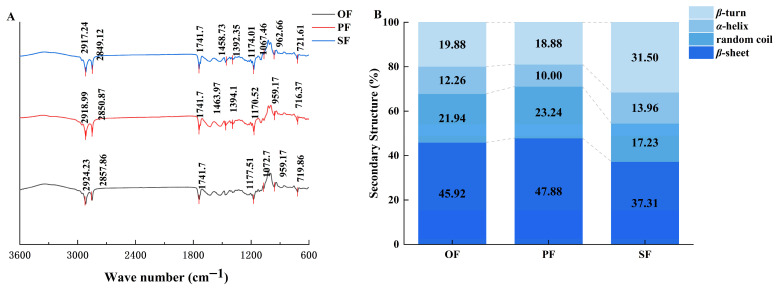
(**A**) Figures of infrared spectra of different oil residue parts of oils smelted at 160 °C; (**B**) Figure of protein secondary structure content of different parts of oils smelted at 160 °C. OF, omental fat; PF, perirenal fat; SF, subcutaneous fat.

FTIR spectroscopy was employed to analyze the chemical composition and structural features of the fats residues. The spectra (Figure 3A) revealed distinct absorption bands characteristic of lipid and protein components. Key bands included the C–H stretching vibration of cis-olefins near 3009 cm^−1^, indicating unsaturated fatty acids, and the C=O stretching vibration of free fatty acids near 1711 cm^−1^ [12]. The absorption at 1654 cm^−1^ corresponds to both protein amide groups and cis C=C bonds, providing structural insights related to the *IVs* discussed earlier [13].

To further elucidate the microstructural basis for the observed oil yield differences (Section 2.1), the protein secondary structure in the residues was quantified. Deconvolution of the amide I band (Figure 3B) showed that SF residue possessed a higher α-helix content (13.96%) and a lower random coil content (17.23%) compared to PF and OF. A higher α-helix to random coil ratio is indicative of a more stable and ordered protein conformation [14,15].

**Figure 4:**
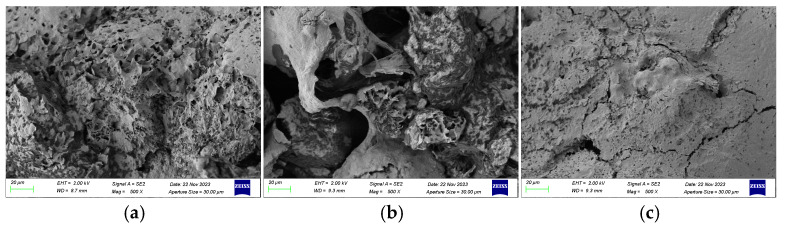
SEM of connective tissue of different parts of tallow residue smelted at 160 °C. (**a**) OF, omental fat; (**b**) PF, perirenal fat; (**c**) SF, subcutaneous fat.

This structural stability directly explains the macroscopic processing properties. The stable protein network in SF likely contributed to maintaining a compact, non-porous microstructure during rendering (as seen in SEM, Figure 4c), which physically hindered lipid release and resulted in a lower oil yield. Conversely, the less ordered protein structures (higher random coil) in OT and PT residues were associated with the formation of looser, porous matrices (Figure 4a,b), facilitating the higher oil yields observed. Thus, FTIR analysis provides a molecular-level rationale linking protein conformational stability to the distinct rendering efficiencies of tallow from different anatomical regions.

### 2.5. Microstructural Characterization of Residues by SEM

SEM of fats residues smelted at 160 °C are presented in Figure 4. Clear structural distinctions were observed among residues originating from different anatomical parts. Residues derived from abdominal tissue and PF displayed a relatively loose microstructure, characterized by ruptured cell walls, numerous pores, and distinct lamellar formations. When considered alongside the results of the protein secondary structure analysis (Figure 4), these observations indicate that the instability of protein conformations in abdominal tissue and PF, combined with heat-induced membrane rupture, promoted the efficient release of oil bodies during the smelting process.

In contrast, residues obtained from SF exhibited a smooth surface with a compact and dense structure containing few pores, which likely restricted lipid release. These observations are consistent with the findings of Zhou et al. [10], who reported that greater intercellular porosity in smelted beef tallow enhances oil yield by facilitating lipid migration through interconnected microstructural networks.

### 2.6. Volatile Flavor Components

The volatile compounds tentatively identified across all sample types are reported in the Supplementary Material (SM, Table S2) while their sum on the basis of chemical classes is summarized in Table 2.

In the raw state (OF, PF, and SF), all fat fractions were dominated by hydrocarbons (52.70–71.87%), which is consistent with the native triglyceride matrix and the lipid-soluble volatile composition of unrendered adipose tissue. Following smelting, a pronounced shift in volatile composition was observed. The relative abundance of hydrocarbons decreased markedly in the rendered oils (1.81–37.63%), while aldehydes became the predominant class of compounds, particularly in perirenal tallow (PT, 84.00%) and omental tallow (OT, 75.40%). This universal transformation is consistent with established lipid oxidation pathways, whereby thermal energy during rendering promotes the degradation of hydrocarbon precursors, such as fatty acid alkoxyl radicals, into a range of aldehydes. These aldehydes are key contributors to the characteristic “tallow” aroma, commonly described as fatty, fried, and nutty [16].

In contrast, the subcutaneous fraction (ST) exhibited a distinct and comparatively less transformed volatile profile. Even after refining, ST retained a substantial proportion of hydrocarbons (37.63%) and developed a lower aldehyde content (39.24%) than PT and OT. This reduced susceptibility to thermal-oxidative flavor development is consistent with earlier findings in this study, which demonstrated higher connective tissue content and lower rendering efficiency for ST. The dense collagen network in ST likely not only restricts fat release but may also alter the local thermal microenvironment or limit reactant mobility, thereby attenuating oxidative flavor-generation pathways that are more pronounced in other fractions. Such structural modulation of flavor chemistry is consistent with reports showing that connective tissue composition directly influences the release and transformation of flavor precursors in meat systems [7].

Among the three fractions, PT emerged as a superior multifunctional material. It exhibited the highest oil yield and greatest hardness and, upon refining, generated the most intense aldehyde-rich aroma profile. This combination of structural robustness and pronounced cooked-fat flavor positions PT tallow as a premium choice for applications requiring both functional stability and strong sensory impact, such as high-quality pastry laminating fats, savory snack formulations, and traditional frying media [17]. Conversely, the ST, characterized by lower yield, softer texture, and a milder, less extensively transformed volatile profile, may be better suited to applications where a subtle flavor contribution is desired or where tallow functions primarily as a structural fat rather than a dominant flavor source, such as in certain emulsified products or as a base for customized flavor enrichment [18]. The OT displayed an intermediate yet highly practical profile, combining satisfactory yield with substantial aldehyde-generation capacity, thereby representing a versatile and efficient option for general culinary and industrial applications.

### 2.7. Principal Component Analysis (PCA) of Volatile Flavor Components

**Figure 5:**
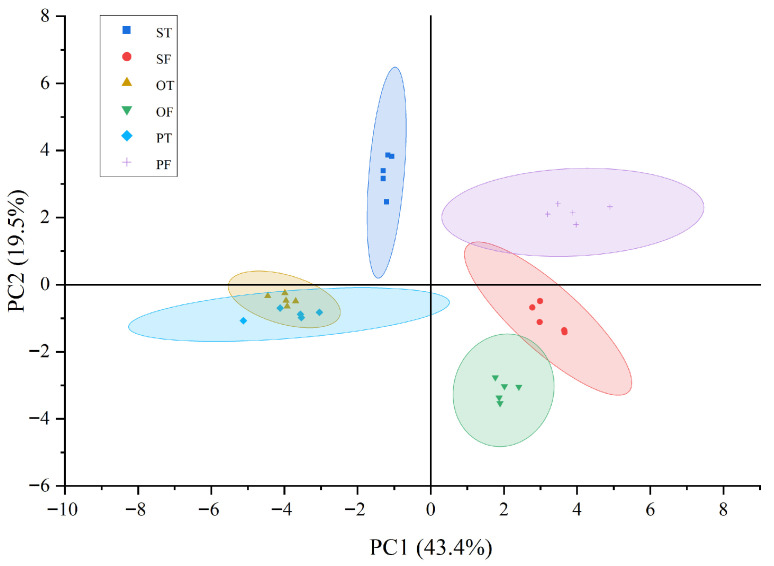
Plot of principal component scores. OF, omental fats; PF, perirenal fats; SF, subcutaneous fats; OT, omental tallow; PT, perirenal tallow; ST, subcutaneous tallow.

**Figure 6:**
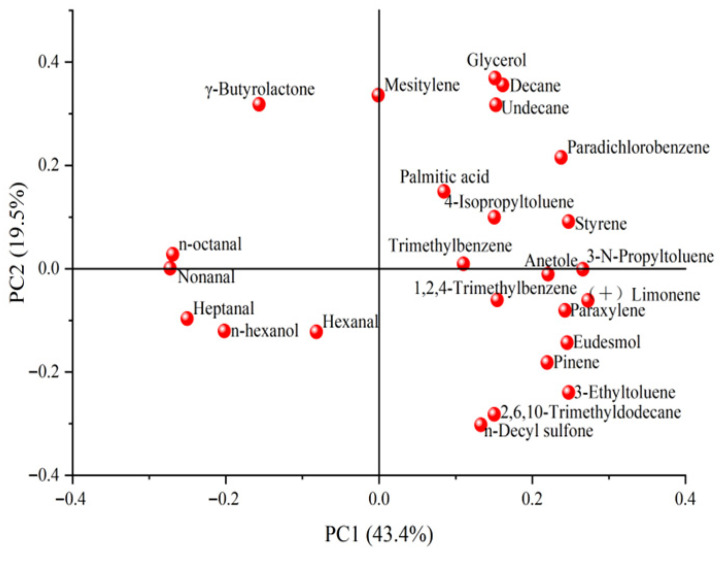
Two-dimensional loading plot of principal components.

In this study, PCA was conducted on 25 volatile compounds with relative contents greater than 1%, identified in both tallow and its corresponding liquid oil. Following the approach of Gu et al. [16], only volatile compounds exceeding 1% in relative abundance were included in the PCA to ensure statistical relevance. The PCA score plot is shown in Figure 5, while the corresponding loading plot is illustrated in Figure 6. Additionally, the eigenvalues and cumulative contribution rates of the correlation matrix are summarized in Table 3, and the eigenvectors of the principal components are presented in Table 4.

As shown in Table 3 and Table 4, the cumulative contribution rate of the first three principal components reached 78.64%, adequately explaining the majority of the information contained within the original 25 variables. The first principal component (PC1), which accounted for 43.41% of the total variance, primarily represented the variability of styrene, 3-ethyltoluene, paradichlorobenzene, (+)-limonene, 3-n-propyltoluene, n-octanal, and heptanal. PC1 exhibited a negative correlation with n-octanal and heptanal, this observation is consistent with the established role of aldehydes as key flavor-active compounds in beef tallow [19], which are primarily generated through the oxidation of polyunsaturated fatty acids, such as linoleic acid. Accordingly, the negative loading of PC1 on these aldehydes reflects their relatively higher abundance in OT and PT, suggesting either a greater availability of precursor fatty acids or an enhanced susceptibility to lipid oxidation in these adipose depots [20]. In contrast, PC1 exhibited positive correlations with styrene, 3-ethyltoluene, paradichlorobenzene, (+)-limonene, 3-n-propyltoluene, and eucalyptol. As shown in Table 2, these compounds were most abundant in PF, which may be related to the unique physiological functions of perirenal fat and the preferential deposition of feed-derived or endogenous lipophilic compounds within this visceral depot [19]. The second principal component (PC2), accounting for 19.49% of the total variance, was mainly associated with decane, undecane, mesitylene, γ-butyrolactone, glycerol, 2,6,10-trimethyldodecane, and di-n-decyl sulfone. PC2 showed positive loadings for decane, undecane, mesitylene, γ-butyrolactone, and glycerol, which may be attributed to the higher proportion of saturated fatty acids in ST. This compositional feature provides abundant substrates for ω-oxidation, thereby facilitating the formation of lactone precursors such as 4-hydroxy acids [21]. Moreover, the close anatomical proximity of ST to muscle tissue may promote the accumulation of metabolic products, including lactic acid, during post-mortem aging [22]. The resulting mildly acidic microenvironment can further enhance intramolecular cyclization and esterification of hydroxy acids during heating and refining, thereby promoting efficient lactone formation. In addition, the relatively lower oxidative stability of ST may suppress the excessive generation of competing oxidation products, such as aldehydes, ultimately accentuating a lactone-dominated, creamy flavor profile. Based on Table 2, these compounds were found to significantly contribute to the flavor profile of SF. The third principal component (PC3), accounting for 15.74% of the total variance, captured the variability of 1,2,4-trimethylbenzene, terphenyl, palmitic acid, n-hexanol, and anethole. Among these, n-hexanol and anethole were negatively correlated with PC3, whereas 1,2,4-trimethylbenzene, terphenyl, and palmitic acid were positively correlated. According to Table 2, these compounds were inferred to play a major role in defining the characteristic flavor profile of SF.

The cumulative contribution rate of the first three principal components reached 78.64%, indicating that they effectively capture most of the variance within the dataset. Based on Table 4, linear regression equations were established for the first three principal components (PC1, PC2, and PC3) to further interpret their relationships and quantify the contribution of each variable to the overall model:

PC1:Y1 = 0.243X_1_ + 0.247X_2_ + 0.219X_3_ + 0.247X_4_ + 0.154X_5_ + 0.161X_6_ + 0.238X_7_ + 0.273X_8_ + 0.266X_9_ + 0.15X_10_ + 0.151X_11_ + 0.152X_12_ + 0.11X_13_ − 0.082X_15_ − 0.251X_16_ − 0.27X_17_ − 0.273X_18_ + 0.085X_19_ − 0.157X_20_ + 0.245X_21_ + 0.202X_22_ + 0.151X_23_ + 0.221X_24_ + 0.133X_25_

PC2:Y2 = −0.081X_1_ + 0.092X_2_ − 0.181X_3_ − 0.239X_4_ − 0.06X_5_ + 0.356X_6_ + 0.216X_7_ − 0.061X_8_ − 0.282X_10_ + 0.1X_11_ + 0.318X_12_ + 0.01X_13_ + 0.336X_14_ − 0.122X_15_ − 0.097X_16_ + 0.028X_17_ + 0.15X_19_ + 0.319X_20_ − 0.143X_21_ − 0.12X_22_ + 0.369X_23_ − 0.302X_25_

PC3:Y3 = 0.264X_1_ − 0.093X_2_ − 0.2X_3_ + 0.007X_4_ + 0.412X_5_ + 0.093X_6_ − 0.028X_7_ − 0.187X_8_ − 0.12X_9_ + 0.289X_10_ + 0.09X_11_ − 0.147X_12_ − 0.305X_13_ + 0.114X_14_ + 0.068X_15_ − 0.007X_16_ − 0.019X_17_ − 0.039X_18_ + 0.348X_19_ + 0.09X_20_ − 0.174X_21_ − 0.276X_22_ + 0.043X_23_ − 0.331X_24_ + 0.281X_25_

In the formula, X_1_~X_25_ represent the standardized variables of the relative contents of 25 components with relative content >1%; each coefficient is the eigenvector of the corresponding substance.

The comprehensive score Y of each sample can be obtained by the calculation of Y = 0.4341Y1 + 0.1949Y2 + 0.1574Y3. Each coefficient represents the contribution rate of the corresponding principal component, and Y1~Y3 are the scores of each principal component. According to the composite Y-score, smelted tallow samples were ranked in the following order: PT > ST > OT (Table 5).

As illustrated in Figure 5, a clear separation was observed between fat and tallow, indicating substantial alterations in volatile flavor compounds before and after the smelting process. This distinction suggests that specific lipid constituents underwent structural transformations during smelting, ultimately modifying the aroma profile of Wagyu tallow. Prior to smelting, animal fat contained abundant natural components, such as esters, aldehydes, alcohols, and short-chain fatty acids, that are capable of releasing intense and characteristic aromas even at relatively low temperatures. However, exposure to elevated temperatures during smelting can induce thermal degradation, oxidation, or volatilization of these compounds, while some short-chain fatty acids may be either volatilized or neutralized, leading to a reduction in natural aromatic intensity. Consequently, the smelted tallow exhibited a milder and more simplified aroma profile compared with raw fat, which retained a greater diversity of original flavor compounds and demonstrated richer aromatic characteristics, as evidenced by its higher overall comprehensive scores.

In addition, the PCA plot revealed a clear separation of SF from PF and OF within the principal component space, indicating substantial compositional differences in their volatile compounds and consequently distinct flavor characteristics. In contrast, PF and OF were positioned in close proximity, reflecting a high degree of similarity in their aromatic compositions and suggesting a convergent flavor profile between the two.

As shown in Figure 5 and Figure 6, PF and OF were primarily distributed within the third quadrant, characterized by volatile compounds such as n-octanal, nonanal, hexanal, and heptanal. These compounds contribute fatty, citrus-like, and grassy aroma notes, indicating that both PF and OF possess a rich and diverse aromatic profile. However, since these compounds exhibited negative correlations with the PC1, both PF and OF recorded relatively low PC1 scores, thereby reducing their overall comprehensive scores.

In contrast, SF was predominantly located within the second quadrant, characterized by γ-butyrolactone as the principal volatile compound. This compound imparts a pronounced buttery aroma with high sensory recognizability. Compared with the other types of fat, SF displayed a more distinctive flavor profile, emphasizing its creamy characteristics and contributing favorably to its overall sensory quality.

## 3. Materials and Methods

### 3.1. Materials and Reagents

Fats samples from different anatomical regions of castrated Wagyu bulls were obtained from Linqing Junbo Foods Co., Ltd., located in Linqing City, Shandong Province, China, a company that operates an integrated breeding farm and slaughterhouse and serves as the sole certified facility for purebred Wagyu beef production. The three types of fats included OF, PF, and SF, corresponding to omental fats, perirenal fats, subcutaneous fats, from 21 Wagyu bulls (28 months, per slaughtering batch) were selected in this experiment, with each type having three samples. The three types of fats, OF, PF, and SF, corresponding to omental, perirenal, and subcutaneous adipose tissues, respectively, were collected from 21 Wagyu bulls (28 months old, per slaughter batch) for this experiment. For each fat type, three representative samples were selected. Hydrochloric acid was procured from Far East Fine Chemical Co., Ltd. (Yantai, China), while anhydrous ethanol was supplied by Angie’s Yeast (Chifeng) Co., Ltd. (Chifeng, China). Glacial acetic acid was obtained from Wuxi Jingke Chemical Co., Ltd. (Wuxi, China). Wijs reagent and sodium thiosulfate standard solution were purchased from Tianjin Komeo Chemical Reagent Co., Ltd. (Tianjin, China). Soluble starch was sourced from Xilong Science Co., Ltd. (Chengdu, China), and potassium iodide was acquired from Tianjin Beilian Fine Chemicals Development Co., Ltd. (Tianjin, China). All chemicals and reagents used in this study were of analytical grade.

### 3.2. Raw Material Handling

Initially, all non-fat tissues were carefully removed from the collected fat samples. The cleaned fat was then cut into uniform cubes measuring 5 cm × 5 cm using a precision knife. After rinsing, the samples were drained to remove excess moisture and subsequently cooled to a controlled temperature range of 10–15 °C. Finally, the chilled fat cubes were ground into a homogeneous paste using a commercial electric meat grinder (Model KK-1123, Zhucheng Ruiheng Food Co., Ltd., Zhucheng, Shandong province, China) equipped with a 4 mm perforated plate to ensure uniform particle size and consistency across all samples.

Samples from each anatomical region (OT, PT, ST) were processed in separate batches. From each batch, a representative 200 g portion of minced tallow adipose tissue (homogenized fat paste) was accurately weighed and transferred into a 1 L beaker. The sample was first melted in a water bath maintained at 70 °C for one hour and then subjected to smelting for 80 min with continuous stirring at 500 revolutions per minute (rpm) using a magnetic heating stirrer. The smelting temperatures were set according to the method described by Kunlun Guo et al. [18] and conducted at four levels: 100 °C, 120 °C, 140 °C, and 160 °C. Following smelting using a digital overhead magnetic stirrer (Model DF-101S, Shanghai Lichen Bangxi Instrument Co., Ltd., Shanghai, China) with precise speed control set at 500 ± 10 rpm, the mixture was filtered through sterile gauze to separate the grease residue, and the clarified tallow was promptly collected for subsequent analyses. All experiments were performed with three biological replicates (n = 3) to ensure reliability, reproducibility, and to account for inherent biological variability.

Oil yield is calculated according to Equation (1).

(1) $$X=\frac{m}{200}\times 100\%$$

In the Formula (1), *X* is the oil yield, expressed as a percentage (%); *m* is the mass of the refined tallow, expressed in grams (g).

### 3.3. Determination of IVs

Approximately 0.6–0.7 g of tallow was accurately weighed into a 500 mL conical flask. A solvent mixture of HPLC-grade cyclohexane and glacial acetic acid (20 mL, 1:1, *v*/*v*) was added to dissolve the sample, followed by the addition of 25 mL of Wijs reagent. The solution was thoroughly mixed and kept in the dark for 1 h to ensure complete reaction. Subsequently, 20 mL of potassium iodide solution and 150 mL of distilled water were added. The resulting mixture was titrated with a 0.1 mol/L sodium thiosulfate standard solution until the yellow color nearly disappeared. After introducing a 0.5% (*w*/*w*) starch indicator, titration was continued until the blue coloration completely faded. The volume of sodium thiosulfate solution consumed was recorded, and distilled water was used as a blank control. Each measurement was conducted in triplicate, and the mean value was calculated. The IVs was determined using the Wijs method in accordance with the China National Standard GB/T 5532-2022 [23].

The *IVs* was calculated according to Equation (2).

(2) $$W=\frac{\left(V_{1}-V_{2}\right)\times C\times 12.69}{m_{1}}$$

In Equation (2), *W* represents the *IV* in grams per 100 g (g/100 g); *V*_1_ is the volume of standard sodium thiosulfate solution consumed by the sample (mL); *V*_2_ is the volume of standard sodium thiosulfate solution consumed by the blank control (mL); *C* denotes the concentration of the standard sodium thiosulfate solution (mol/L); and *m*_1_ is the mass of the oil sample (g).

### 3.4. Hardeness

Tallow samples from three distinct anatomical regions of Wagyu beef were placed in a beaker and heated in a water bath at 55 °C until completely melted. The liquefied tallow was then poured into cylindrical containers with a diameter of 4 cm and a liquid level height of 1 cm. The filled containers were subsequently refrigerated at 4 °C for 45 min to produce uniformly solidified tallow samples of consistent thickness for subsequent analyses.

Texture profile analysis (TPA, TA-XT Plus, Stable Micro Systems, Surrey, UK) was conducted using a penetration probe (P/2, diameter 2 mm) under conditions similar to those described by Zeng et al. [5]. The test parameters were as follows: pre-test speed of 2 mm/s, test speed of 2 mm/s, penetration depth of 5 mm, and trigger force of 5 g. Each uniformly prepared sample was analyzed in technical quadruplicate to ensure the accuracy and reproducibility of the measurements.

### 3.5. FTIR

The prepared oil residue was cut into meat columns measuring 0.3 × 0.3 × 0.2 cm. The samples were dehydrated three times using 95% ethanol (Taiyuan Qingxu Kangjiu Pharmaceutical Excipients Welfare Factory, Taiyuan, China), with each dehydration step lasting 30 min, subsequently, to ensure complete removal of any residual water, the samples were subsequently treated with 99.7% ethanol (Anhydrous Ethanol, Tianjin Fuyu Fine Chemical Co., Ltd., Tianjin, China) for 15 min to remove residual moisture and fix the protein components in the tallow residue. Following dehydration, the samples were dried in an oven at 30 °C for 4 h. The dried residues were ground into a fine powder using an agate mortar and pestle to prevent contamination. Each sample was ground for approximately 5 min, until the powder could pass through a 150-mesh nylon sieve (particle size ≤100 μm). The sieved powder was then homogenized and stored in a desiccator until FTIR analysis. 2.5 mg of the ground powder was then evenly placed into a clean sample cell, approximately, ensuring uniform distribution without visible agglomeration or voids. To prevent cross-contamination, the sample cell was thoroughly wiped with ethanol between successive tests.

FTIR spectra were acquired using a Nicolet iS5 spectrometer (Thermo Scientific, Madison, WI, USA) equipped with an ATR accessory (iD7, Thermo Scientific, Madison, WI, USA), following the method of Pu et al. [24] with slight modifications. Measurements were performed using a single-bounce zinc selenide crystal. Dried powder samples were evenly spread onto the crystal plate of a FTIR spectrometer and scanned within the wavenumber range of 400–4000 cm^−1^ at a resolution of 4 cm^−1^, with 32 scans accumulated per sample. Each biological sample was analyzed in technical triplicate to ensure reproducibility of the method. The resulting spectra were processed to eliminate interference peaks caused by water vapor and carbon dioxide. Quantification of the protein secondary structure was performed based on the amide I band using OMNIC and PeakFit software (version 4.12) for spectral deconvolution and curve fitting. The final processed spectra represented the infrared absorption characteristics of the connective tissue residues and the protein secondary.

### 3.6. SEM

The microstructure of the solid residues was examined using SEM. Residue blocks (approximately 0.3 × 0.3 × 0.2 cm) from different anatomical regions were first dehydrated with 95% and 99.7% ethanol, as described in Section 3.5 (FTIR). To preserve the native microstructure and avoid artifacts caused by ice crystal formation, the dehydrated blocks were not immersed in water. Instead, they were directly transferred onto a sample stage and rapidly frozen in a liquid nitrogen slush for 10 min.

The frozen samples were then subjected to sublimation drying in a freeze dryer (LGJ-10, Beijing Songyuan Huaxing Technology Development Co., Ltd., Beijing, China) for 48 h to ensure complete removal of moisture and residual solvents. The lyophilized samples were mounted on aluminum stubs using double-sided conductive carbon tape and sputter-coated with a 10 nm-thick layer of gold using a high-resolution sputter coater (Q150R S, Quorum Technologies, East Sussex, UK) to ensure conductivity.

Microstructural observations were conducted with a field-emission SEM (GeminiSEM 500, ZEISS, Oberkochen, Germany). Images were acquired at magnifications of 500×, 1000×, and 2000×, using an accelerating voltage of 3.0 kV, a standard setting for biological and soft materials to minimize charging and sample damage, under high-vacuum conditions [25].

### 3.7. Volatile Organic Compounds

The method was carried out following Ahamed et al. [26], with slight modifications. HS-SPME was conducted using a DVB/CAR/PDMS (50/30 μm) fiber (Supelco, Bellefonte, PA, USA). For each analysis, 2.0 g of solid fat or tallow sample was placed in a 20 mL headspace vial containing a magnetic stir bar and 1.0 g of NaCl was added to promote the salting-out effect and enhance the release of volatile compounds. Prior to extraction, 10 μL of an internal standard solution (2-methyl-3-heptanone, 50 μg/mL in methanol) was added using a microsyringe for semi-quantitative analysis. The vial was equilibrated at 60 °C for 15 min with continuous stirring at 500 rpm, after which the fiber was exposed to the headspace for 30 min. A blank vial containing only NaCl and the internal standard was processed alongside each batch of samples to monitor system background and potential fiber contamination. The adsorbed volatiles were thermally desorbed in the GC inlet at 250 °C for 2 min in splitless mode, followed by a split ratio of 4:1. A deactivated glass inlet liner (without packing) was used, and the fiber was conditioned at 250 °C for 30 min prior to first use each day.

Gas chromatography-Mass spectrometry (GC/MS, Agilent 7890A/5975C, Agilent Technologies, Santa Clara, CA, USA) conditions: The analysis was conducted using an HP-5MS capillary column (60 m × 0.25 mm, 0.25 μm film thickness) with helium as the carrier gas at a constant flow rate of 1.0 mL/min. The oven temperature program was set as follows: an initial temperature of 40 °C held for 1 min, increased at a rate of 5 °C/min to 250 °C, and maintained for 5 min.

Analysis was performed using an HP-5MS column for compound separation. Mass spectrometric detection was carried out using electron ionization at 70 eV, with an ion source temperature of 230 °C and a transfer line temperature of 250 °C. The mass range was scanned from *m*/*z* 35 to 500, with a solvent delay of 3.0 min. Compound identification was conducted by comparing mass spectra with the NIST library, requiring a match factor ≥ 800.

For semi-quantitative analysis, the relative peak areas of individual compounds were used directly without correction. Each sample was analyzed in technical triplicate to ensure reproducibility. The data were processed using the Modified Z-score method, followed by removal of extreme high and low values. The relative content of each compound was calculated based on the ratio of its peak area to that of the internal standard (2-methyl-3-heptanone).

To determine the LRI of each analyte, a homologous series of a n-alkane solution (C7-40, HJ894-2017, ANPEL, Shanghai, China; 5 mg/L) was used in hexane under the same chromatographic conditions as those used for the samples, the tolerance criteria (e.g., ±20 units depending on the basis of column) used for comparing experimental LRI values with literature/database references on the HP-5MS/DB-5 column. The LRI was calculated with Equation (3) [27].

(3) $$LRI=\left(100\times n\right)+\left(100\times z\right)\times \frac{tr\left(compound\right)-tr\left(n\right)}{tr\left(N\right)-tr\left(n\right)}$$

In Equation (3), tr represents retention time; n and N represent the numbers of carbon atoms in the eluting alkanes before and after the product is produced, z represents the discrepancy in the number of carbon atoms in the smaller and larger alkanes.

### 3.8. Data Analysis

All experiments were conducted with three independent biological replicates (n = 3). Data are presented as mean ± SD.

For parameters measured with multiple instrumental readings (e.g., texture in quadruplicate, GC-MS in quintuplicate), the value for each biological replicate was calculated as the average of its corresponding technical replicates.

For GC-MS-derived volatile compound data, the peak area of each compound was first normalized to the peak area of the internal standard (2-methyl-3-heptanone) within the same run to obtain a corrected response. The relative abundance of each compound was then calculated based on these corrected responses. For FTIR spectral data, vector normalization was applied to minimize the effects of baseline drift and sample concentration differences.

The significance of differences in physicochemical indices (oil yield, hardness, *IVs*) among different anatomical regions and refining temperatures was assessed by two-way analysis of variance, followed by Duncan’s multiple range test for post hoc comparison. A *p*-value of less than 0.05 was considered statistically significant. Statistical differences (*p* < 0.05) are indicated by different superscript letters within a row (lowercase) or column (uppercase), whereas identical letters denote no significant difference.

To visualize the overall differences in volatile compound profiles and to identify key flavor compounds contributing to these differences, PCA was performed on the normalized, internal standard-corrected relative abundance data. The reliability of the PCA model was evaluated by the cumulative variance contribution rate of the principal components.

Data processing was carried out using Microsoft Excel 2019, including recording raw data, calculating the averages of technical replicates for each biological sample, and computing final group means and SDs. All statistical analyses were performed using SPSS software (version 26.0, IBM, New York, NY, USA), and graphs were prepared with Origin 2022.

## 4. Conclusions

This study systematically elucidates the substantial variations in technological properties and volatile flavor profiles among Wagyu beef tallow derived from three key anatomical regions (OF, PF, and SF), thereby providing a scientific foundation for their part-specific utilization.

With respect to processing suitability, the oil yield was found to be strongly influenced by both refining temperature and anatomical origin. Rendering at 160 °C produced the maximum yield across all regions, with PF consistently exhibiting the highest yield, followed by OF and SF. These regional differences were fundamentally linked to their distinct microstructural characteristics. FTIR and SEM observations revealed that SF, characterized by a more stable protein secondary structure, formed a compact residue during rendering, thereby restricting oil release. In contrast, the less stable protein conformations in OF and PF resulted in loose, porous residues, which facilitated more efficient oil migration.

Beyond processing traits, a clear region-specific differentiation was also identified in the volatile flavor profile. OF and PF displayed a similar flavor signature dominated by aldehydes responsible for fatty and citrusy notes. In contrast, SF demonstrated a distinctive creamy aroma, primarily attributed to γ-butyrolactone. It should be noted that the identification of volatile compounds in this study was based on mass spectral library matching. While a high similarity threshold was applied, future research would benefit from the additional confirmation provided by Linear Retention Indices (LRIs) using n-alkane standards analyzed under identical chromatographic conditions. This would further solidify the identification of key aroma-active compounds and is a recommended direction for enhancing the analytical precision in the characterization of Wagyu tallow flavors.

Collectively, these findings enable a move beyond a one-size-fits-all approach. The integrated assessment of yield, stability, and flavor supports the precision utilization of distinct Wagyu tallow fractions: PF and OF are recommended for applications requiring high yield and pronounced flavor intensity, whereas SF, with its unique creamy aroma, is ideal for specialized products where this distinctive note is desired. This part-specific strategy promises to optimize both the economic value and sensory quality of Wagyu tallow in advanced food formulations.

## Figures and Tables

**Table 1:** Oil yield and residue quality of different parts of tallow at different smelting temperatures.

Index	Temperature (°C)		Sample	
		OT	PT	ST
oil yield/%	100	56.78 ± 1.73 ^Ab^	61.62 ± 5.11 ^Ab^	32.38 ± 2.58 ^Aa^
	120	59.93 ± 3.90 ^ABb^	64.02 ± 3.21 ^ABb^	38.98 ± 2.11 ^ABa^
	140	60.72 ± 2.46 ^ABb^	63.62 ± 5.32 ^ABb^	54.62 ± 3.46 ^ABa^
	160	64.58 ± 1.00 ^Bb^	72.42 ± 3.85 ^Bb^	59.03 ± 0.60 ^Ba^
Residue/g	100	18.27 ± 3.9 ^Ba^	32.9 ± 4.33 ^Ba^	100.90 ± 4.40 ^Bb^
	120	15.77 ± 0.75 ^ABa^	23.54 ± 3.85 ^ABa^	81.53 ± 3.94 ^ABb^
	140	8.20 ± 1.00 ^ABa^	10.03 ± 2.16 ^ABa^	39.10 ± 1.98 ^ABb^
	160	5.43 ± 0.67 ^Aa^	9.53 ± 1.02 ^Aa^	23.20 ± 0.97 ^Ab^

a~b indicates the difference between different sites is significant (*p* < 0.05); A~B indicates the difference between different temperatures is significant (*p* < 0.05). OT, omental tallow; PT, perirenal tallow; ST, subcutaneous tallow.

**Table 2:** Types of volatile substances and their proportion in the total.

Category	OF%	PF%	SF/%	OT/%	PT/%	ST/%
Hydrocarbons	52.7	60.33	71.87	6.67	1.81	37.63
Aldehydes	30.11	19.01	12.49	75.4	84	39.24
Ketones	0	0	3.04	1.96	1.66	0
Acids	1.28	2.43	0.36	0.77	2.67	8.21
Alcohols	4.04	14.74	8.94	12.11	8.59	12.59
Ester	4.86	0.57	0.69	2.57	1.08	1.55
Ethers	0.17	1.52	2.27	0.34	0.06	0
Phenols	4.43	0	0	0	0	0
Heterocyclic and others	2.41	1.4	0.34	0.18	0.13	0.78
Total	100	100	100	100	100	100

OF, omental fats; PF, perirenal fats; SF, subcutaneous fats; OT, omental tallow; PT, perirenal tallow; ST, subcutaneous tallow.

**Table 3:** The eigenvalues and cumulative contribution rates of the correlation matrix.

Principal Component	Eigenvalue	Contribution/%	Cumulative Contribution Rate/%
1	10.85	43.41	43.41
2	4.87	19.49	62.89
3	3.93	15.74	78.63

**Table 4:** Principal component eigenvectors.

Eigenvector	Principal Component 1	Principal Component 2	Principal Component 3
Paraxylene X_1_	0.243	−0.081	0.264
Styrene X_2_	0.247	0.092	−0.093
α-Pinene X_3_	0.219	−0.181	−0.2
3-Ethyltoluene X_4_	0.247	−0.239	0.007
1,2,4-Trimethylbenzene X_5_	0.154	−0.06	0.412
Decane X_6_	0.161	0.356	0.093
p-Dichlorobenzene X_7_	0.238	0.216	−0.028
((+)-Limonene X_8_	0.273	−0.061	−0.187
3-N-propyltoluene X_9_	0.266	0	−0.12
2,6,10-Trimethyl dodecane X_10_	0.15	−0.282	0.289
4-Isopropyltoluene X_11_	0.151	0.1	0.09
Undecane X_12_	0.152	0.318	−0.147
Bis(trimethyl)benzene X_13_	0.110	0.01	−0.305
Homotrimethylbenzene X_14_	0	0.336	0.114
Hexanal X_15_	−0.082	−0.122	0.068
HeptaldehydeX_16_	−0.251	−0.097	−0.007
Octanal X_17_	−0.27	0.028	−0.019
Nonanal X_18_	−0.273	0	−0.039
Hexadecanoic acid X_19_	0.085	0.15	0.348
Gamma-butyrolactone X_20_	−0.157	0.319	0.09
Eucalyptol X_21_	0.245	−0.143	−0.174
Hexyl alcohol X_22_	0.202	−0.120	−0.276
Glycerin X_23_	0.151	0.369	0.043
Anisole X_24_	0.221	0	−0.331
Di-n-decyl sulfone X_25_	0.133	−0.302	0.281

**Table 5:** Overall score and ranking of different parts of tallow.

Part	Y Composite Score/Points	Ranking/Place
OF	−1.9718	6
PF	−1.2209	5
SF	0.0612	4
OT	0.13338	3
PT	0.84323	1
ST	0.4227	2

OF, omental fats; PF, perirenal fats; SF, subcutaneous fats; OT, omental tallow; PT, perirenal tallow; ST, subcutaneous tallow.

## Data Availability

The original contributions presented in this study are included in the article. Further inquiries can be directed to the corresponding authors.

## Supplementary Materials

The following supporting information can be downloaded at: https://www.mdpi.com/article/10.3390/molecules31030426/s1, Table S1: Hardness of Tallow duplicate samples; Table S2: volatile components and relative content of fats [28,29,30,31,32,33,34,35,36,37,38,39,40,41,42,43,44,45].

## Abbreviations

The following abbreviations are used in this manuscript:

SEM	Scanning electron microscopy
FTIR	Fourier infrared
PCA	Principal component analysis
*IVs*	Iodine values
