# Supplementary material for: Processing Suitability and Flavor Profiles of Wagyu Beef Tallow from Different Anatomical Regions
Source: Molecules. 2026 Jan 26;31(3):426. doi: 10.3390/molecules31030426 (PMC12898603; doi:10.3390/molecules31030426)
Supplement: Supplementary file 1 [file molecules-31-00426-s001.zip › molecules-4006233-supplementary.pdf]

Supplementary materials

Table S1. Hardness of Tallow duplicate samples

Tallow	Duplicate samples	Hardness/g
ST	1	286.3
ST	2	265.85
ST	3	239.7
ST	4	228.04
AVERAGE±SD	ST (F)	254.97±22.68
OT	1	894.66
OT	2	869.93
OT	3	836.56
OT	4	817.69
AVERAGE:	OT (F)	854.71±29.7
PT	1	1073.24
PT	2	1077.68
PT	3	1049.78
PT	4	1035.19
AVerage:	PT (F)	1058.97±17.35

Table S2. Volatile components and relative content of fats

Number	Volatile flavor components	Chemical formula	CAS	Relative content (%)						RT(min)						Lit. LRI (HP-5MS/DB-5)	Calc. LRI (Average)	Source of LRI
				OT	PT	ST	OF	PF	SF	OT	PT	ST	OF	PF	SF			
1	1-Chlorohexane	$C_6H_{13}Cl$	544-10-5	-	-	-	2.23	-	-	-	-	-	3.81	-	-		759.938	
2	Ethylbenzene	C_8H_{10}	100-41-4	-	-	-	6.52	0.83	-	-	-	-	5.93	5.7	-	856	859.36	[27]
3	p-Xylene	C_8H_{10}	106-42-3	0.29	-	1.24	6.52	2.87	1.71	5.06	-	6.43	6.16	5.92	5.5	857	859.316	[27]
4	Styrene	C_8H_8	100-42-5	-	-	-	1.59	4.25	2.04	-	-	-	6.72	6.52	6.15	890.5	887.783	[29]
5	*α-Pinene*	$C_{10}H_{16}$	80-56-8	-	-	-	0.57	0.34	1.31	-	-	-	7.95	7.79	7.52	936.1	930.721	[29]
6	n-Propylbenzene	C_9H_{12}	103-65-1	-	-	-	0.52	0.57	0.63	-	-	-	8.53	8.4	8.17		949.352	
7	3-Ethyltoluene	C_9H_{12}	620-14-4	0.56	0.21	-	4.73	3.01	4.39	8.2	8.69	-	8.77	8.65	8.43	960	954.86	[30]
8	1,2,4-Trimethylbenzene	C_9H_{12}	95-63-6	-	-	2.13	7.93	3.34	-	-	-	10.49	9.71	8.85	-	993	1002.569	[31]

9	Decane	$C_{10}H_{22}$	124-18-5	0.33	0.17	2.94	0.87	2.94	1.44	9.53	9.87	9.82	9.92	9.83	9.68	1004	992.132	[31]
10	p-Dichlorobenzene	$C_6H_4Cl_2$	106-46-7	-	-	1.98	1.88	6.15	2.55	-	-	10.15	10.25	10.16	10.01		1003.579	
11	(+)-Limonene	$C_{10}H_{16}$	5989-27-5	-	-	-	4	5.1	6.58	-	-	-	10.79	10.71	10.58	1023	1020.029	
12	3-N-propyltoluene	$C_{10}H_{14}$	1074-43-7	0.07	-	-	0.48	1.18	0.8	11.18	-	-	11.47	11.41	11.3		1042.904	
13	2,6,10-Trimethyl dodecane	$C_{15}H_{32}$	3891-98-3	-	-	-	2.49	0.52	0.45	-	-	-	13.31	13.27	13.21	1100	1106.65	[31]
14	4-Isopropyltoluene	$C_{10}H_{14}$	99-87-6	0.27	-	-	0.55	1.76	-	12.28	-	-	12.49	12.44	-	1024.3	1077.836	[29]
15	2,7-Dimethyloctane	$C_{10}H_{22}$	1072-16-8	0.4	-	-	-	-	-	11.51	-	-	-	-	-		1048.489	
16	Undecane	$C_{11}H_{24}$	1120-21-4	0.23	0.12	0.95	-	1.66	1.09	12.76	12.91	12.89	-	12.89	12.83	1099	1092.829	[32]
17	2-Nonyl ethylene oxide	$C_{11}H_{22}O$	17322-97-3	0.09	-	-	-	-	-	15.6	-	-	-	-	-		1190.492	
18	2,3'-Dioxopentane	$C_7H_{13}NO_3$	104313-51-1	0.22	0.29	-	-	-	-	12.59	12.74	-	-	-	-		1086.432	
19	6-Methyloctadecane	$C_{19}H_{40}$	10544-96-4	0.16	0.11	-	-	-	-	15.79	15.85	-	-	-	-	1196	1198.386	[31]
20	Cyclooctatetraene	C_8H_8	629-20-9	0.15	-	-	-	-	-	5.77	-	-	-	-	-		857.387	
21	Bis(trimethyl)benzene	C_9H_{12}	526-73-8	0.36	-	2.87	-	-	9.13	10.22	-	8.84	-	-	9.44	1058	994.033	[33]

22	1-methyl-2-propylbenzene	$C_{10}H_{14}$	1074-17-5	-	-	-	0.33	-	0.71	-	-	-	11.93	-	11.4		1053.581	
23	Tetradecane	$C_{14}H_{30}$	629-59-4	-	-	-	2.34	-	-	-	-	-	12.92	-	-	1089	1094.809	[27]
24	1,2-Epoxyoctane	$C_8H_{16}O$	2984-50-1	-	0.51	-	-	-	-	-	4.04	-	-	-	-		774.092	
25	Homotrimethylene	C_9H_{12}	108-67-8	-	0.22	13.36	-	2.04	3.02	-	10.54	9.6	-	10.5	10.36	965	1007.365	[31]
26	4-Chlorooctane	$C_8H_{17}Cl$	999-07-5	-	0.08	-	-	-	-	-	11.01	-	-	-	-		1032.063	
27	(3E)-3-prop-2-enylidenecyclobutene	C_7H_8	52097-85-5	-	-	0.54	-	0.65	-	-	-	3.35	-	3.41	-		733.477	
28	trans-β-methylstyrene	C_9H_{10}	637-50-3	-	-	0.98	-	-	-	-	-	10.89	-	-	-		1028.121	
29	1,2-Dimethyl-4-ethylbenzene	$C_{10}H_{14}$	934-80-5	-	-	-	-	1.58	2.6	-	-	-	-	11.63	11.53	1078	1050.789	[31]
30	4-methyl-decane	$C_{11}H_{24}$	2847-72-5	-	-	-	-	0.42	2.13	-	-	-	-	11.7	11.61	1024	1053.253	[32]
31	1,2-dimethyl-3-ethylbenzene	$C_{10}H_{14}$	933-98-2	-	-	-	-	1.03	1.56	-	-	-	-	12.25	12.16		1071.321	
32	2,6-dimethyldecane	$C_{12}H_{26}$	13150-81-7	-	-	-	-	0.21	-	-	-	-	-	14.55	-		1152.817	
33	3-ethyl-5-(2-ethylbutyl)octadecane	$C_{26}H_{54}$	55282-12-7	-	-	-	-	0.31	-	-	-	-	-	14.79	-		1161.428	
34	n-Octadecane	$C_{18}H_{38}$	26741-18-4	-	-	-	-	0.99	-	-	-	-	-	14.99	-		1168.604	

35	Dodecane	$C_{12}H_{26}$	112-40-3	-	-	-	-	1.73	-	-	-	-	-	15.84	-	1199	1199.103	[32]
36	Ethylbenzene	C_8H_{10}	100-41-4	-	-	-	-	-	1.08	-	-	-	-	-	5.26	837	835.028	[27]
8	m-Xylene	C_8H_{10}	108-38-3	-	-	-	-	-	3.15	-	-	-	-	-	5.5	843	845.55	[27]
38	Dimethyloctane	$C_{10}H_{22}$	2051-30-1	-	-	-	-	-	0.48	-	-	-	-	-	8.75		960.604	
39	Hinokiene	$C_{10}H_{16}$	3387-41-5	-	-	-	-	-	1.99	-	-	-	-	-	8.81	973	962.819	[29]
40	2-Ethyltoluene (o-toluene)	C_9H_{12}	611-14-3	-	-	-	-	-	4.41	-	-	-	-	-	9		968.591	
41	P-cymene	$C_{10}H_{14}$	535-77-3	-	-	-	-	-	0.68	-	-	-	-	-	10.45	1022	1013.666	[29]
42	Indane	C_9H_{10}	496-11-7	-	-	-	-	-	1.05	-	-	-	-	-	10.77		1024.179	
43	2-methyldecane	$C_{11}H_{24}$	6975-98-0	-	-	-	-	-	0.47	-	-	-	-	-	11.71	1055	1055.059	[27]
44	1-methyl-4-n-propylbenzene	$C_{10}H_{14}$	1074-55-1	-	-	-	-	-	0.48	-	-	-	-	-	11.78		1057.359	
45	3-Methyldecane	$C_{11}H_{24}$	13151-34-3	-	-	-	-	-	0.47	-	-	-	-	-	11.92	1069	1061.958	[31]
46	3-Methyleicosane	$C_{21}H_{44}$	6418-46-8	-	-	-	-	-	0.68	-	-	-	-	-	13.35		1109.76	
47	3-Methylundecane	$C_{12}H_{26}$	1002-43-3	-	-	-	-	-	0.62	-	-	-	-	-	14.95	1173	1167.169	[31]

48	Cyclohexene	$C_9H_{13}NO_2$	80255-20-5	2.51	-	-	-	-	-	9.26	-	-	-	-	-		976.488	
49	Hexanal	$C_6H_{12}O$	66-25-1	-	17.32	8.42	12.19	5.25	7.88	-	4.25	4.09	4.45	4.14	3.53	799.9	777.292	[29]
50	Heptanal	$C_7H_{14}O$	111-71-7	16.04	10.75	5.96	6.38	1.3	2.15	6.19	6.92	6.83	7.03	6.86	6.54	902	897.893	[29]
51	(Z) -2-Heptenal	$C_7H_{12}O$	57266-86-1	-	-	-	0.63	-	-	-	-	-	8.64	-	-	960	957.655	[32]
52	n-Octylaldehyde	$C_8H_{16}O$	124-13-0	13.22	11	5.81	2.4	3.73	-	9.64	9.97	9.93	10.02	9.94	-	1002.8	995.93	[29]
53	Nonanal	$C_9H_{18}O$	124-19-6	15.12	13.86	6.91	2.38	3.16	-	12.92	13.07	13.04	13.07	13.04	-	1103.3	1098.357	[29]
54	trans-2-nonanal	$C_9H_{16}O$	18829-56-6	0.11	3.35	-	0.74	-	-	14.22	14.32	-	14.71	-	-	1162.2	1143.973	[29]
55	Decanal	$C_{10}H_{20}O$	112-31-2	0.6	0.49	-	0.16	0.26	-	15.97	16.03	-	16.04	16.02	-	1205.4	1205.27	[29]
56	Trans-2-hexenal	$C_6H_{10}O$	6728-26-3	1.14	0.98	-	-	-	-	4.57	5.58	-	-	-	-	817	804.998	[29]
57	Trans-2-heptenal	$C_7H_{12}O$	18829-55-5	2.94	0.41	-	-	-	-	8.06	4.86	-	-	-	-	806.5	817.492	[29]
58	(E, E) -2,4-heptadienal	$C_7H_{10}O$	5/3/4313	0.78	1.71	-	-	-	-	9.43	9.78	-	-	-	-	1011.5	981.652	[29]
59	trans-2-octenal	$C_8H_{14}O$	2548-87-0	4.62	3.23	-	-	-	-	11.08	11.31	-	-	-	-	1060.2	1034.63	[29]
60	(trans) -4-nonenal	$C_9H_{16}O$	2277-16-9	0.13	-	-	-	-	-	12.69	-	-	-	-	-		1087.254	

61	（1R，2S，5S）-2-(1R, 2S, 5S) -2-methyl-5-(3-oxoprop-1-en-2-yl) cyclopentane-1-carbaldehyde	$C_{10}H_{14}O_2$	5951-57-5	0.17	0.16	-	-	-	-	13.52	13.65	-	-	-	-		1120.524	
62	(Z) -4-decenal	$C_{10}H_{18}O$	21662-09-9	0.19	0.16	-	-	-	-	15.73	15.79	-	-	-	-	1203	1196.233	[34]
63	(E, E) -2,4-nonadienal	$C_9H_{14}O$	5910-87-2	0.07	2.43	-	-	-	-	16.2	9.61	-	-	-	-	1215.8	1211.771	[29]
64	(Z) -2-decenal	$C_{10}H_{18}O$	2497-25-8	4.6	-	-	-	-	-	17.57	-	-	-	-	-		1259.909	
65	Undecanal	$C_{11}H_{22}O$	112-44-7	0.14	0.11	-	-	-	-	18.83	18.85	-	-	-	-	1306.5	1304.629	[29]
66	Trans-2,4-decadienal	$C_{10}H_{16}O$	25152-84-5	1.67	0.97	-	-	-	-	19.08	19.1	-	-	-	-	1317.6	1311.984	[29]
67	2-Undecylenal	$C_{11}H_{20}O$	2463-77-6	1.82	2.31	-	-	-	-	20.34	20.36	-	-	-	-	1363	1358.809	[35]
68	(E, E) -2,4-dodecadienal	$C_{12}H_{20}O$	21662-16-8	0.4	-	-	-	-	-	18.46	-	-	-	-	-		1291.181	
69	Trans-2-pentenal	C_5H_8O	1576-87-0	-	0.35	-	-	-	-	-	3.34	-	-	-	-	744	731.015	[37]
70	(Z) -4-Heptenal	$C_7H_{12}O$	6728-31-0	-	0.13	-	-	-	-	-	6.79	-	-	-	-	886	901.458	[38]
71	2-Heptenal	$C_7H_{12}O$	57266-86-1	-	3.98	-	-	-	-	-	8.54	-	-	-	-	960	954.617	[32]
72	4-Methyl-3-cyclohexenal	$C_8H_{12}O$	7560-64-7	-	0.2	-	-	-	-	-	10.83	-	-	-	-		1026.15	
73	2, 4-nonadienal	$C_9H_{14}O$	4/3/675	-	0.0	-	-	-	-	-	16.	-	-	-	-	1215.8	1213.879	[29]

			0		6						26							
74	10-Octadecenal	$C_{17}H_{32}O_2$	56554-92-8	-	0.05	-	-	-	-	-	17.16	-	-	-	-		1245.502	
75	Trans-2-decenal (2-Decenal, (E)-)	$C_{10}H_{18}O$	3913-81-3	-	5.17	-	-	-	-	-	17.23	-	-	-	-	1263.4	1247.962	[29]
76	Dodecaldehyde	$C_{12}H_{24}O$	112-54-9	-	0.05	-	-	-	-	-	21.53	-	-	-	-		1401.564	
77	Phenylacetaldehyde	C_8H_8O	122-78-1	-	-	1.04	-	-	-	-	-	11.17	-	-	-	1045.9	1037.319	[29]
78	2-heptanone	$C_7H_{14}O$	110-43-0	0.87	-	-	-	-	-	5.84	-	-	-	-	-	891.7	860.456	[29]
79	Hexahydro-3-methylenebenzofuran-2 (3H) -one	$C_9H_{12}O_2$	53387-38-5	0.51	-	-	-	-	-	10.54	-	-	-	-	-		1016.623	
80	2-nonanone	$C_9H_{18}O$	821-55-6	0.18	0.21	-	-	-	-	12.53	12.67	-	-	-	-	1092.5	1084.297	[29]
81	3-nonen-2-one	$C_9H_{16}O$	14309-57-0	0.1	0.08	-	-	-	-	14	14.11	-	-	-	-		1137.029	
82	4-Methyl-2-hexanone	$C_7H_{14}O$	105-42-0	-	0.94	-	-	-	-	-	6.62	-	-	-	-		894.651	
83	Trans-3-octen-2-one	$C_8H_{14}O$	18402-82-9	-	0.05	-	-	-	-	-	11.09	-	-	-	-		1034.691	
84	Ethyl cyclopentenolone	$C_7H_{10}O_2$	21835-01-8	-	0.16	-	-	-	-	-	13.47	-	-	-	-		1114.065	
85	2-Decanone	$C_{10}H_{20}O$	693-54-9	-	0.12	-	-	-	-	-	15.66	-	-	-	-	1190	1192.644	[27]
86	2-Dodecanone	$C_{12}H_{24}O$	6175-49	-	0.0	-	-	-	-	-	23.	-	-	-	-		1509.275	

			-1		1						7							
87	2-Methyl-3-octanone	$C_9H_{18}O$	923-28-4	-	-	-	-	-	1.52	-	-	-	-	-	9.21	975	974.97	[27]
88	Methyl heptenone	$C_8H_{14}O$	110-93-0	-	-	-	-	-	0.92	-	-	-	-	-	9.3		977.704	
89	1,2,4, -Benzenetricarboxylic acid	$C_{11}H_{10}O_6$	54699-35-3	-	-	-	0.21	-	-	-	-	-	4.33	-	-		791.938	
90	Hexadecanoic acid	$C_{16}H_{32}O_2$	3/10/1957	-	0.05	1.03	0.85	0.44	0.29	-	33.41	33.7	33.72	33.71	33.7	2168.4	2160.424	[29]
91	Stearic acid	$C_{18}H_{36}O_2$	4/11/1957	0.04	-	-	-	-	-	33.69	-	-	-	-	-	2172.4	2162.765	[29]
92	Hexanoic acid	$C_6H_{12}O_2$	142-62-1	0.61	1.56	-	-	-	-	9.1	9.49	-	-	-	-	996.4	977.552	[29]
93	Butyric acid	$C_4H_8O_2$	107-92-6	-	0.07	-	-	-	-	-	3.93	-	-	-	-	799	767.323	
94	Pentanoic acid	$C_5H_{10}O_2$	109-52-4	-	0.12	-	-	-	-	-	6.47	-	-	-	-	808.3	888.075	[29]
95	3-heptenoic acid	$C_7H_{12}O_2$	29901-85-7	-	0.33	-	-	-	-	-	12.26	-	-	-	-		1073.127	
96	2-hexyl-1-cyclopropylacetic acid	$C_{11}H_{20}O_2$	35936-15-3	-	0.25	-	-	-	-	-	15.03	-	-	-	-		1170.039	
97	Nonanoic acid	$C_9H_{18}O_2$	112-05-0	-	0.14	-	-	-	-	-	17.8	-	-	-	-	1277	1267.99	[32]
98	B-terpineol acetic acid	$C_{12}H_{20}O_2$	10198-23-9	-		4.86	-	-	-	-	-	10.7	-	-	-		905.103	
99	Isovaleric acid	$C_5H_{10}O_2$	503-74-	-	-	-	-	1.	-	-	-	-	-	5.2	-	860.4	834.152	[29]

			2					31						4				
100	N-butyl acrylate	$C_7H_{12}O_2$	141-32-2	-	-	-	0.36	-	-	-	-	-	6.91	-	-		905.103	
101	9,12-Octadecadienoic acid (9Z, 12Z) -phenylmethyl ester	$C_{25}H_{38}O_2$	47557-83-5	-	-	-	0.57	-	-	-	-	-	9.12	-	-	1154	972.236	[29]
102	Vinyl hexanoate	$C_8H_{14}O_2$	3050-69-9	-	-	-	2.51	-	-	-	-	-	9.46	-	-		982.564	
103	γ-butyrolactone	$C_4H_6O_2$	96-48-0	0.73	0.18	1.11	-	0.41	-	6.58	7.26	7.2	-	7.23	-	916.48	909.343	[38]
104	1-Methyl-4- (1-methylvinyl) cyclohexanol acetate	$C_{12}H_{20}O_2$	10198-23-9	0.83	0.28	-	-	-	-	10.45	10.74	-	-	-	-		1018.43	
105	Benzyl oleate	$C_{24}H_{40}O_2$	55130-16-0	0.21	0.12	-	-	-	-	10.96	5.77	-	-	-	-		943.904	
106	γ-Heptalactone	$C_7H_{12}O_2$	105-21-5	0.16	0.17	-	-	-	-	14.41	14.51	-	-	-	-	1130	1149.587	[34]
107	g-Octalactone	$C_8H_{14}O_2$	104-50-7	0.09	0.09	-	-	-	-	17.48	17.52	-	-	-	-		1257.449	
108	Tris (2-chloropropyl) phosphate	$C_8H_{18}Cl_3O_4F$	13674-84-5	0.11	-	-	-	-	-	30.62	-	-	-	-	-		1947.376	
109	Butyl isooctyl phthalate	$C_{20}H_{30}O_4$	84-78-6	0.04	0.04	-	-	-	-	33.81	33.82	-	-	-	-		2170.011	
110	4-Hexadecyl ester	$C_{22}H_{44}O_2$	78-21-7	-	-	-	0.58	-	-	-	-	-	9.54	-	-		984.994	
111	Formic acid [(phenylmethyl) sulfoxide] -1,1-dimethylethyl ester (9CI)	$C_7H_{12}O$	57266-36-1	-	-	-	-	-	0.55	-	-	-	-	-	8.31		947.631	
112	Benzyl eicosanoate	$C_{27}H_{46}O_2$	77509-0	-	0.1	-	-	-	-	-	11.	-	-	-	-		1038.633	

			4-7		4						21							
113	trans-1,2-cyclopentadiol	$C_5H_{10}O_2$	5057-99-8	-	0.06	-	0.18	-	-	-	3.96	-	3.6	-	-		758.092	
114	2-ethylhexane-1-thiol	$C_8H_{18}S$	7341-17-5	-	-	-	1.2	-	-	-	-	-	9.05	-	-		970.109	
115	4-Ethylcyclohexanol	$C_8H_{16}O$	4534-74-1	-	-	-	0.36	-	-	-	-	-	9.84	-	-	1032	994.107	[40]
116	Cineole	$C_{10}H_{18}O$	470-82-6	-	-	-	0.74	0.89	1.76	-	-	-	10.87	10.8	10.67	1032	1024.507	[34]
117	2-Hexyl-1-octanol	$C_{14}H_{30}O$	19780-79-1	-	-	-	0.23	-	-	-	-	-	12	-	-		1064.586	
118	2,4-Dimethylcyclohexanol	$C_8H_{16}O$	69542-91-2	0.2	0.09	-	0.63	-	-	10.64	10.92	-	12.08	-	-		1038.743	
119	3-Methyl-1,2-cyclopentanediol	$C_6H_{12}O_2$	27583-37-5	0.06	0.06	-	-	-	-	4.26	5.32	-	-	-	-		812.645	
120	1-Hexanol	$C_6H_{14}O$	111-27-3	1.47	0.99	-	-	-	0.76	5.15	6.03	-	-	-	5.59	837	849.496	[32]
121	1α, 2β, 3β, 4α-cyclopentadiol	$C_5H_{10}O_4$	14003-71-5	0.12	-	-	-	-	-	5.7	-	-	-	-	-		854.318	
122	2,7-Dimethyl-2,6-octadiene-1-ol	$C_{10}H_{18}O$	22410-74-8	0.11	-	-	-	-	-	7.23	-	-	-	-	-		914.824	
123	1-Heptanol	$C_7H_{16}O$	111-70-6	2.99	2.98	-	-	-	-	8.56	8.97	-	-	-	-	968.6	961.453	[29]
124	1-Octen-3-ol	$C_8H_{16}O$	3391-86-4	2.3	-	-	-	-	-	8.86	-	-	-	-	-	877	964.33	[34]
125	3-Methyl-1,6-heptadien-3-ol	$C_8H_{14}O$	34780-6	0.2	-	-	-	-	-	9.3	-	-	-	-	-	973	978.919	[39]

			9-3	2						4								
126	Rosin alcohol	$C_{10}H_{16}O$	6712-79-4	0.14	-	0.9	-	-	-	10.78	-	11.62	-	-	-		1038.305	
127	Perillyl alcohol	$C_{10}H_{16}O$	536-59-4	0.07	-	-	-	-	-	11.13	-	-	-	-	-		1036.005	
128	2-Decene-1-ol	$C_{10}H_{20}O$	22104-80-9	0.12	0.05	-	-	-	-	11.79	11.97	-	-	-	-		1060.644	
129	Octanol	$C_8H_{18}O$	111-87-5	1.97	1.78	-	-	-	-	11.86	12.04	-	-	-	-	1071.5	1062.944	[35]
130	Z) -phenol, 2-methyl-5- (1-methylvinyl) -2-cyclohexene-1-ol, cis-menthol-1,8-diene-6-ol	$C_{10}H_{16}O$	1197-06-4	0.26	-	-	-	-	-	12.01	-	-	-	-	-	1115	1064.915	[45]
131	(E) -3-octen-1-ol	$C_8H_{16}O$	20125-85-3	0.12	-	-	-	-	-	12.1	-	-	-	-	-		1067.871	[42]
132	Cis-3-decanol	$C_{10}H_{20}O$	10340-22-4	0.09	-	-	-	-	-	20.74	-	-	-	-	-	1365	1372.802	[42]
133	n-Pentanol	$C_5H_{12}O$	71-41-0	-	2.05	-	-	-	-	-	3.57	-	-	-	-	765.5	745.169	[35]
134	1-nonen-3-ol	$C_9H_{18}O$	21964-44-3	-	0.04	-	-	-	-	-	6.51	-	-	-	-		889.829	
135	Erythritol	$C_4H_{10}O_4$	149-32-6	-	-	1.96	-	-	-	-	-	9	-	-	-		968.591	
136	Glycerol	$C_3H_8O_3$	56-81-5	-	-	5.31	-	7.45	1.7	-	-	9.34	-	9.46	9.09		977.602	
137	(1R, 2S, 4R, 5R) -rel-1,7,7-trimethylbicyclo [2.2.1] heptane-2,5-diol	$C_{10}H_{18}O_2$	10359-41-8	-	-	0.86	-	-	-	-	-	10.79	-	-	-		1024.836	
138	Diglycerol	$C_6H_{14}O_5$	627-82-	-	-	-	-	2.	-	-	-	-	-	9.1	-		971.932	

			7					28						1				
139	(2R, 3R) - (-) -2,3-butanediol	$C_4H_{10}O_2$	24347-58-8	-	-	-	-	-	1.61	-	-	-	-	-	3.3	806	728.554	[42]
140	Diglycerol	$C_6H_{14}O_5$	627-82-7	-	-	-	-	-	1.35	-	-	-	-	-	8.89		965.249	[28]
141	Anisole	$C_{10}H_{12}O$	104-46-1	0.07	0.06	-	0.14	1.1	1.82	18.25	18.29	-	18.29	18.28	18.27		1284.715	[28]
142	Allyl methyl ether	$C_7H_{12}O$	14289-96-4	0.22	-	-	-	-	-	6.04	-	-	-	-	-	830	869.224	[44]
143	p-Methylphenol	C_7H_8O	106-44-5	-	-	-	3.66	-	-	-	-	-	12.18	-	-	1077.4	1070.499	[44]
144	5- (a-Phenylethyl) aminooxahydrazine	$C_{10}H_{13}N_3O_2$	93-95-8	-	-	-	0.27	-	-	-	-	-	7.68	-	-		928.493	
145	Cyclobarbital	$C_{12}H_{16}N_2O_3$	52-31-3	-	0.02	-	0.35	-	-	-	20.47	-	7.75	-	-		1363.114	[36]
146	Di-n-decyl sulfone	$C_{20}H_{42}O_2S$	111530-37-1	0.05	0.02	-	1.29	0.24	0.27	21.27	28.37	-	13.94	13.91	14.18		1315.714	[36]
147	8-Bromoquinoline	C_9H_6BrN	16567-18-3	-	-	-	0.08	-	-	-	-	-	15.38	-	-		1182.598	[36]
148	3-Phenylethylbenzonitrile	$C_{15}H_{13}N$	34176-91-5	0.04	-	-	-	0.77	-	7.92	-	-	-	3.45	-		836.785	
149	L-mannose	$C_6H_{12}O_5$	35810-56-1	0.06	-	-	-	-	-	8.98	-	-	-	-	-		967.983	[28]
150	2,3-Dimethylpiperidine	$C_7H_{15}N$	5347-68-2	-	0.07	-	-	-	-	-	17.03	-	-	-	-		1240.935	
151	O-decylhydroxylamine	$C_{10}H_{23}NO$	29812-7	-	-	0.5	-	-	-	-	-	15.	-	-	-		1199.935	[28]

9-1	6	84	

Note: CAS is the registration number of chemical substances by Chemical Abstracts Service.

Reference:

[27]Pranata, A.W.; Yuliana, N.D.; Amalia, L.; et al. Volatilomics for Halal and Non-Halal Meatball Authentication Using Solid-Phase Microextraction–Gas Chromatography–Mass Spectrometry. Arabian J. Chem. 2021, 14, 103146.

[28]Umano, K.; Hagi, Y.; Nakahara, K.; Shoji, A.; Shibamoto, T. Volatile Chemicals Identified in Extracts from Leaves of Japanese Mugwort (Artemisia Princeps Pamp.). J Agric Food Chem 2000, 48, 3463 – 3469, doi:10.1021/jf0001738.

[29]Babushok, V.I.; Linstrom, P.J.; Zenkevich, I.G. Retention Indices for Frequently Reported Compounds of Plant Essential Oils. J Phys Chem Ref Data 2011, 40, doi:10.1063/1.3653552.

[30]Kotowska, U.; Żalikowski, M.; Isidorov, V.A. HS-SPME/GC–MS Analysis of Volatile and Semi-Volatile Organic Compounds Emitted from Municipal Sewage Sludge. Environ. Monit. Assess. 2012, 184, 2893–2907. https://doi.org/10.1007/s10661-011-2158-8.

[31]Faussone, G.C.; Cecchi, T. Chemical Recycling of Plastic Marine Litter: First Analytical Characterization of the Pyrolysis Oil and of Its Fractions and Comparison with a Commercial Marine Gasoil. Sustainability 2022, 14, 1235. https://doi.org/10.3390/su14031235.

[32]He, Y.; Chen, L.; Liu, W.; et al. Comparative Analysis of the Volatile Components in Chinese Breast Milk from Three Regions. Food Sci. Biotechnol. 2023, 32, 903–909.

[33]Zenkevich, I.G.; Nosova, V.E. Gas-Chromatographic Retention Indices in GC/MS Identification of Alkyl Dichlorophosphates, Dialkyl Chlorophosphates, and Their Thio Analogues. J. Anal. Chem. 2019, 74, 1421–1436. https://doi.org/10.1134/S1061934819140120.

[34]Sheibani, E.; Duncan, S.E.; Kuhn, D.D.; et al. Changes in Flavor Volatile Composition of Oolong Tea after Panning during Tea Processing. Food Sci. Nutr. 2016, 4, 456–468. https://doi.org/10.1002/fsn3.307.

[35]Hu, B.B.; Yin, W.T.; Yu, T.; et al. A Novel Approach to Sesame Oil Aroma Generation: Synergy of Enzyme-Modified Lipids and the Maillard Reaction. Food Chem. 2025, 492, 144985. https://doi.org/10.1016/j.foodchem.2025.144985.

[36]Vasiliki, S.; Nikos, D.; Zacharias, K.; et al. Analysis of the Essential Oil Composition of Eight Anthemis Species from Greece. J. Chromatogr. A 2006, 1104, 313–322.

[37]Yasar, S. Volatile Constituents of Taxus baccata L. Leaves from Western and Southern Turkey. Asian J. Chem. 2013, 25, 9123–9125. https://doi.org/10.14233/ajchem.2013.15038.

[38]Tang, K.; Chen, C.; Liu, Y.; et al. Comprehensive Characterization of Aroma Profile of “Glutinous Rice” Flavor in Pandanus amaryllifolius Roxb. Using Hs-Spme-Gc-O-Ms and Hs-Gc-Ims Technology Coupled with Oav. 2024. https://doi.org/10.3390/foods14060935.

[39]Lan, H.; Lin, X.; Ma, H.; Lu, L.; Liao, W.; Wang, Y.; Chen, Y.; Li, C. Formation of Aroma Characteristics in Roasted Camellia oleifera

Seeds. Foods 2026, 15, 87. https://doi.org/10.3390/foods15010087.
[40]Woerdenbag, H.J.; Bos, R.; Salomons, M.C.; Hendriks, H.; Pras, N.; Malingré, T.M. Volatile constituents of Artemisia annua L. (Asteraceae). Flavour Fragr. J. 1993, 8, 131 - 137, https://doi.org/10.1002/ffj.2730080303.
[41]Gaumann, T. Gas Chromatography. Annu. Rev. Phys. Chem. 1965, 16, 125–146. https://doi.org/10.1146/annurev.pc.16.100165.001013. Nurhayat, T.; Betul, D.; L, S.C.; et al. Chemical Composition and Antifungal Activity of Arnica longifolia, Aster hesperius, and Chrysothamnus nauseosus Essential Oils. J. Agric. Food Chem. 2007, 55, 8430–8435.
[42]Tabanca, N., Demirci, B., Crockett, S. L., Başer, K. H., Wedge, D. E. Chemical composition and antifungal activity of Arnica longifolia, Aster hesperius, and Chrysothamnus nauseosus essential oils. J. Agric. Food Chem. 2007, 55, 8430–8435. https://doi.org/10.1021/jf071379c
[43]Bin, X., Li, W., Yaqin, G. Comparative Analysis of Nutrient Components of Yak Milk from Different Regions. Anim. Husb. Feed. Sci. 2010, 2, 26-48.
[44]Esmaeili, A.; Nematollahi, F.; Rustaiyan, A.; Moazami, N.; Masoudi, S.; Bamasian, S. Volatile constituents ofAchillea pachycephala,A. oxyodonta andA. biebersteinii from Iran. Flavour Fragr. J. 2005, 21, 253 - 256, https://doi.org/10.1002/ffj.1571.
[45]Guido, F.; Luigi, P.C.; Ivano, M. Differences in the Fragrances of Pollen, Leaves, and Floral Parts of Garland (Chrysanthemum coronarium) and Composition of the Essential Oils from Flowerheads and Leaves. J. Agric. Food Chem. 2003, 51, 2267–2271.

Same squence with the ref. in the main text.

Lit. LRI means, literature of the linear retention indexes.

Calc. LRI means, calculated retention indexes.
